# Community engagement and the social context of targeted malaria treatment: a qualitative study in Kayin (Karen) State, Myanmar

**DOI:** 10.1186/s12936-017-1718-y

**Published:** 2017-02-14

**Authors:** Kate Sahan, Christopher Pell, Frank Smithuis, Aung Kyaw Phyo, Sai Maung Maung, Chanida Indrasuta, Arjen M. Dondorp, Nicholas J. White, Nicholas P. J. Day, Lorenz von Seidlein, Phaik Yeong Cheah

**Affiliations:** 1 The Ethox Centre, Nuffield Department of Population Health, University of Oxford, Old Road Campus, Oxford, OX3 7LF UK; 2 Centre for Social Science and Global Health, University of Amsterdam, Amsterdam, The Netherlands; 3 Medical Action Myanmar, Yangon, Myanmar; 4 Centre for Tropical Medicine, Nuffield Department of Clinical Medicine, University of Oxford, Oxford, OX3 7LF UK; 5 Mahidol-Oxford Tropical Medicine Research Unit (MORU)-Faculty of Tropical Medicine, 420/6 Rajvithi Road, Bangkok, 10400 Thailand; 6 Amsterdam Institute for Global Health and Development, Amsterdam, The Netherlands

**Keywords:** Community engagement, Mass drug administration, Malaria, Elimination

## Abstract

**Background:**

The spread of artemisinin-resistance in *Plasmodium falciparum* is a threat to current global malaria control initiatives. Targeted malaria treatment (TMT), which combines mass anti-malarial administration with conventional malaria prevention and control measures, has been proposed as a strategy to tackle this problem. The effectiveness of TMT depends on high levels of population coverage and is influenced by accompanying community engagement activities and the local social context. The article explores how these factors influenced attitudes and behaviours towards TMT in Kayin (Karen) State, Myanmar.

**Methods:**

Semi-structured interviews were conducted with villagers from study villages (N = 31) and TMT project staff (N = 14) between March and July 2015.

**Results:**

Community engagement consisted of a range of activities to communicate the local malaria situation (including anti-malarial drug resistance and asymptomatic malaria), the aims of the TMT project, and its potential benefits. Community engagement was seen by staff as integral to the TMT project as a whole and not a sub-set of activities. Attitudes towards TMT (including towards community engagement) showed that developing trusting relationships helped foster participation. After initial wariness, staff received hospitality and acceptance among villagers. Offering healthcare alongside TMT proved mutually beneficial for the study and villagers. A handful of more socially-mobile and wealthy community members were reluctant to participate. The challenges of community engagement included time constraints and the isolation of the community with its limited infrastructure and a history of conflict.

**Conclusions:**

Community engagement had to be responsive to the local community even though staff faced time constraints. Understanding the social context of engagement helped TMT to foster respectful and trusting relationships. The complex relationship between the local context and community engagement complicated evaluation of the community strategy. Nonetheless, the project did record high levels of population coverage.

## Background

Although the greatest malaria burden lies in sub-Saharan Africa, the spotlight has recently returned to South East Asia, where artemisinin resistant *Plasmodium falciparum* is increasingly prevalent [1–4]. The spread of multidrug resistant *P. falciparum* is one of the greatest threats to malaria control [5], and, for the World Health Organization (WHO), elimination of *P. falciparum* is an urgent priority in the greater Mekong subregion (GMS) [6]. If not eliminated, resistant parasites could spread across Asia to Africa, as has happened with other resistant strains in the past [7].

In response, efforts to eliminate *P. falciparum* in the GMS have intensified. One initiative is targeted malaria treatment (TMT), sometimes referred to as Targeted Malaria Elimination, an integrated approach that combines mass drug administrations (MDAs) with conventional anti-malarial control activities, such as strengthening a network of village health workers (VHWs) to provide appropriate case management, and distribute long-lasting insecticide-treated bed nets. In spite of the mixed results of previous MDA efforts, this approach is now viewed as a potential tool to reduce the spread of artemisinin resistance in *P. falciparum* [7–9]. This entails administering a curative anti-malarial dose to all individuals within a village irrespective of malaria infection without reliance on diagnostic tests. Extensive research is currently underway to examine the potential of TMT for *P. falciparum* elimination in areas of suspected or proven artemisinin resistance across the GMS, such as Kayin State, Myanmar. Stemming drug resistance in Myanmar, which has the highest prevalence of malaria in the GMS and is known as the “gateway for drug resistance” to malaria, offers important strategic gains [4, 10].

The success of MDAs is predicated on high population coverage [9]. Although questions remain about the precise level of coverage required to interrupt malaria transmission, this along with the efficacy of the drug regimen and local malarial epidemiology are crucial for the success of MDA [8]. To promote coverage (and adherence), previous programmes of anti-malarial MDAs and MDAs for other diseases have undertaken a range of activities, such as employing local people, including VHWs and other field workers, getting village leader support, providing health education, and involving communities [11]. Broadly, such activities have been referred to as community engagement.

Community engagement is, however, a commonly used term in international health research meaning its definition and function differs widely. Some researchers prioritize community engagement to promote the success of projects, in terms of study objectives or health outcomes, whereas others focus on its value in promoting ethical research practice [12, 13]. Moreover, if community engagement is mentioned at all in the reporting of medical research, few details are generally given. In the case of mass anti-malarial administration, although researchers describe community engagement as crucial to promote population coverage [7, 8, 14], the local social context is also highly relevant. For example, local understandings of medication might make the taking of medication in the absence of symptomatic disease unacceptable [8].

To date, little research has addressed specifically how community engagement and social and cultural factors influence coverage and adherence to a programme of mass anti-malarial drug administration [15, 16]. No such research has specifically addressed this topic in Myanmar or focused on MDA. In response, a qualitative study of TMT participants and staff was conducted alongside a TMT project undertaken by Medical Action Myanmar (MAM) [17] in Kayin State, Myanmar. The results of the qualitative study are presented in this article which explores the community engagement that was undertaken as part of TMT and its impact, along with that of the local social and cultural context, on attitudes and behaviours towards TMT.

## Methods

### Setting

Targeted malaria treatment was conducted in 10 villages in Kyaingseikgyi, the most southern township of the Kayin State, which is located in eastern Myanmar near the Thai border. Normally a 12 h drive from Yangon, flood water, armed road blocks and mountainous terrain can impede and prevent access to the area. Previous decades saw clashes between government armed forces and state-level armed groups in this region, but in recent years the situation has stabilized. Migration is common, historically to escape armed conflict, and to access markets and festivals, or find employment. Other migration routes are followed by shopkeepers who trade in one village but live in another, children from remote villages who attend regional boarding schools in other villages, and workers who travel to Thailand and return to their villages once a year. The infrastructure in this township is very limited. Most villages are remote and only accessible by motorbike or on foot and health facilities are few and far between. The high mobility of the population [18, 19] makes this region a challenging setting for MDA [20].

In 2012, MAM started to set up a network of VHWs in the remote villages of this township. These VHWs were trained to provide a package of basic health care including the diagnosis and treatment of malaria. In addition to this basic health care package, a referral system was set up, so that severe and complicated patients could be referred to government hospitals. Transport fees, which are high due to the remoteness of this area and, therefore, a limiting factor for villagers, were paid by MAM.

### Targeted malaria treatment and mass anti-malarial administration

**Fig. 1:**
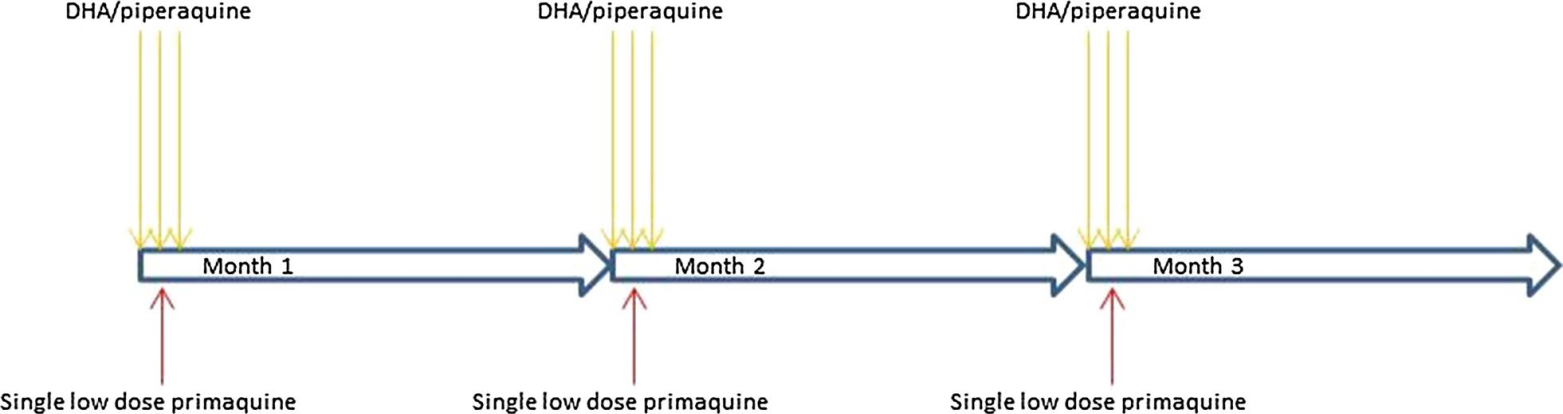
A schematic representation of treatment regimen in TMT [7]

Targeted malaria treatment project activities were first introduced through many small group discussions between the villagers and staff. This was followed by a census to establish the potentially eligible population and subsequently three rounds of anti-malarial administrations, the first and last of which were respectively preceded and followed by representative prevalence surveys using a highly sensitive quantitative PCR (qPCR) [21]. The three rounds of MDA were conducted 1 month apart, each round consisting of three daily doses of dihydroartemisinin/piperaquine combined with a single low dose primaquine (0.25 mg/kg) on the first day of each round (see Fig. 1). An average of 90% of the population in the TMT villages received at least one full round of MDA.

### Qualitative data collection

The objectives of this qualitative study were to explore the way community engagement and social context impacted attitudes and behaviours with respect to the delivery and success of the TMT project. The study population of interest was the Kayin community, including villagers from TMT villages, local village authority figures and locally-employed staff working on the TMT project. Insights into the community were also sought from other non-local TMT staff. One-to-one semi-structured interviews were conducted from March to July 2015. Respondents were selected based on a mixture of snowballing, diversity, and pragmatic sampling approaches, which were dependent on access to field sites. A total of 31 villagers and 14 staff were interviewed. Villagers were identified from the TMT census; some had consented to participation in TMT, others had refused. Staff were identified by the operations coordinator. Villager interviews were conducted by a Kayin fieldworker with prior experience of qualitative research. At the time of the study, foreigners were prohibited from entering this region, making it impossible for the expatriate members of the research team to accompany the fieldworker to the villages. However, the fieldworker was extensively debriefed upon returning from the research site and took photographs of study villages and transit routes, as well as field notes. Project staff were interviewed in Yangon by members of the research team (PYC and KS). All interviews were audio-recorded. Interviews with villagers were conducted in Kayin dialects and interviews with staff were in English or Burmese with the help of an interpreter All interviews were recorded and transcribed, and translated into English, if necessary.

Interview guides for villagers explored five areas relating to community engagement and the social context of the TMT: (1) personal history (migration, poverty, conflict); (2) perceived health problems (malaria/fever concepts); (3) perceptions of taking drugs for malaria prevention (treatment-seeking behaviour); (4) involvement in the TMT programme; and (5) opinions on community engagement in the programme. With staff members, the following topics were discussed: (1) roles and responsibilities in TMT; (2) descriptions of community engagement activities; and (3) operational or social challenges of TMT. Interviewee characteristics are found in Table 1.

**Table 1:** Interviewee characteristics

Designation	Ethnicity	Gender		Total
		Male	Female	
Villager	Kayin	15	13	28
Village leader	Kayin	3	0	3
TMT staff	Kayin	4	1	5
	Burmese	4	1	5
	Shan	2	0	2
	Thai	0	1	1
Total		29	16	45

Interview transcripts and images were supported by profiles of each TMT village (n = 10) compiled by TMT project staff. These profiles covered population density, physical characteristics of villages tending towards malaria transmission, villager behavioural characteristics, such as migration and mobility practices, and existing malaria prevention and treatment efforts such as VHWs and use of long-lasting insecticidal bed nets.

### Qualitative data analysis

The approach to data analysis combined inductive and deductive elements: analytical categories were developed from the initial research questions and also emerged during the analysis process. Using a software package for the purpose (QSR NVivo 10), these categories were operationalized as codes in a flexible coding scheme. Independently, two authors (KS and CP) conducted line-by-line coding of all interview transcripts. They, along with a third author (PYC) discussed extensively the outcome of the coding, with particular emphasis on discrepancies in the application of the pre-determined codes and the introduction of novel categories. The content of the codes and subsequent discussions were used to develop the themes that are presented below and to explore patterns of these themes across the transcripts/data sources. Photos taken by the fieldworker were used to give those doing analysis a fuller visual account of the research setting and sensitize them to the social and cultural context. However, they did not form a formal part of the analysis.

## Results

This study explored the community engagement activities and associated challenges as well as the local social context of a TMT pilot study in Kayin state, Myanmar. The data illustrate how community engagement was implemented in complex circumstances, with staff facing challenges and local sensitivities associated with a research setting, which has recently experienced prolonged conflict and lacks healthcare infrastructure.

### Engaging the communities

**Fig. 2:**
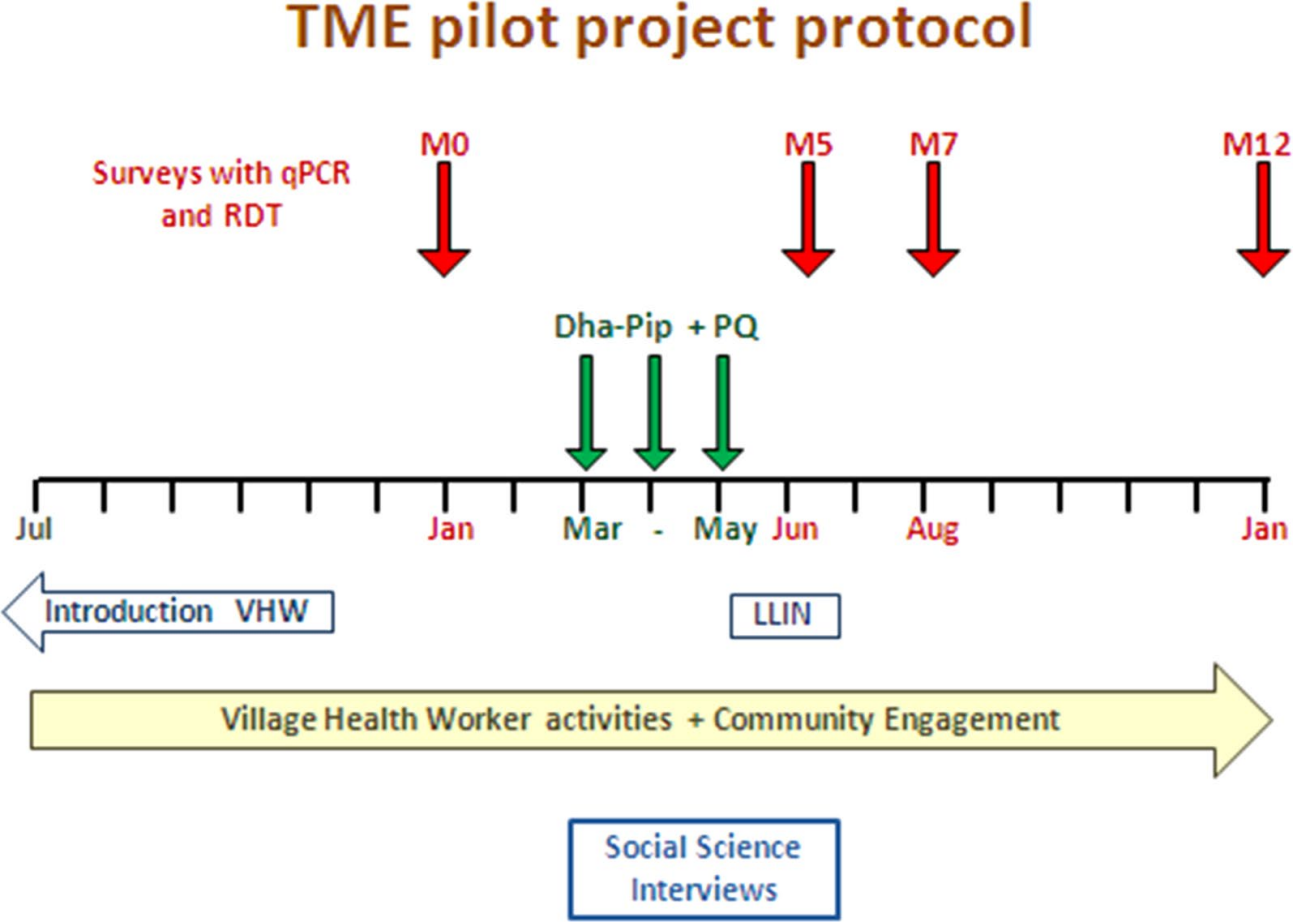
Timing and duration of community engagement activities

The aim of community engagement was to present the TMT study in a manner that would encourage understanding and buy-in among the target communities and result in a high coverage of the TMT interventions. As part of this, staff aimed to promote respect and trust, and allay fears over potential risks. The TMT team, including one community engagement coordinator, and four to six locally hired field assistants conducted the community engagement activities, which are shown in Fig. 2 along with the clinical activities and data collection. Before each drug administration round and every round of blood sampling for qPCR that followed (at months 5, 7 and 12) community engagement activities were conducted. Staff, however, adapted the intensity of community engagement according to villagers’ responses to TMT. Community engagement activities focused on local village authority figures, in addition to villagers themselves.

#### Village authority figures

Senior members of the TMT team discussed the project with village authority figures and institutions to obtain approval. As well as gaining national governance approval, the project underwent multiple approval processes with different levels of government. In Kayin State, approval from both administration and healthcare officials was needed but not granted initially, necessitating repeat visits to the Kayin state and township authorities involved. Approvals were also needed from armed groups, whose road blocks initially stopped staff travelling to TMT villages. Up to three armed groups were named, and staff described how more groups existed and that negotiation was needed with all of them, with or without a formal approval process. Groups engaged with TMT staff by intensive questioning about their motives:

The [armed group] wanted to know, ‘*Why these villages? Why now? Are there any other projects? Who we are, where we are from?*’ (S61, staff member).

One armed group required staff to re-translate study documentation, even though it was already available in the local language. Staff were also filmed by that group. Soldiers attended village community engagement meetings. A special community engagement session was even arranged in their mess. Relations with one group improved because they recognized TMT staff as having previously delivered another health education project in the area. Staff reported that local armed groups were not aggressive, and that they were primarily there to “protect their own ethnic” group. However, government armed forces were felt to be a threat, and even ethnically Burmese staff were wary of them after an incident early in the study:

‘*During the [preliminary prevalence survey], they came around with the guns’*.

(S62, staff member).

Ultimately, staff built trust and rapport with groups: one was given presents by soldiers and another, who had never seen a real gun before, was offered a session of target practice.

Village leaders and secretaries (who acted as liaisons between villagers and the Burmese military, collecting military tax and manpower) or other key individuals, such as teachers, VHWs, traditional healers, monks (one of whom was an astrologer, a highly influential practice in Myanmar) and representatives of armed groups also participated in community engagement activities. Meetings were held to explain TMT and ask for help to gather and persuade villagers. Sometimes, the village leader was not the most authoritative or influential person in the village.

‘*The key person is the monk and he is the astrologer… the head of the villages are not a very good high authority’.*

(S62, staff member).

#### Villagers

Community engagement was intensified prior to the baseline malaria prevalence survey, the three rounds of anti-malarial administration, and the follow-up prevalence surveys (see Fig. 2). Meetings were conducted, either by prior appointment and arranged by authority figures, or opportunistically to maximize numbers: such events involved whole villages, part village ‘clusters’, social groupings, such as women’s groups or youth groups.

*‘When we entered into the village, we went to the places where we saw the people gathering… For example, when we went to the village and found a construction site, we went there because there were more people working together’.*

(S52, staff member).

Village leaders took active roles and encouraged villagers to attend meetings or anti-malarial administration. Staff conducted house-to-house visits and follow-up if people did not return for subsequent doses. Sometimes, to achieve this, villagers were followed into the forest, or transported from remote areas to attend meetings. Staff also had to work around villagers’ schedules: some preferred to take drugs in the early morning before they went to the field or farm, or at midday when they had finished work; others preferred earlier in the evening when it was cooler or directly before they went to bed so that they could sleep off any resulting drowsiness.

During the study, staff managed adverse events and provided treatment for other ailments that any villager experienced. Staff developed strategies to avoid over-treatment: one team member recounted how he had to conceal his supply of intravenous drugs as their popularity with villagers was high but not related to realistic needs.

A TMT logo and branding was developed. This was added to balloons for children, books, wrist bands, T-shirts and umbrellas, which were given to villagers as incentives to pass through each study stage. In addition, snacks and drinks were distributed at meetings or as part of dosing visits. To compensate for loss of earnings, villagers were given 1000 Myanmar Kyats (approximately 1 USD) per dosing visit. Although most villagers did not have ‘daily work’ arrangements, a minority reported to their employers on a daily basis.

When introducing the project, staff emphasized that a high malaria disease burden in the community had been established by specialized blood tests performed in an off-site laboratory, and that they wanted to treat as many people as possible in the village to reduce that burden. Villagers could ask questions about risks and adverse effects of the drug treatment either at community engagement meetings, or anti-malarial administration.

Common questions were noted by staff and disseminated at TMT team update meetings in an effort to respond to community needs and adapt existing approaches. In particular, staff noted that certain scientific concepts (asymptomatic disease, low parasite loads, resistance and targeted treatment), were difficult to explain to villagers, in particular those with little knowledge about healthcare in general and research concepts and with generally little formal education. Among these concepts, the participation of those with asymptomatic disease proved particularly difficult: villagers queried why they should take drugs—and potentially suffer adverse events—when they felt healthy. For this reason, staff felt that basic healthcare education should be given to improve villagers’ understanding of health, disease and medical research. Staff suggested that NGOs with expertise in healthcare education are well placed to do this.

Some staff struggled to easily separate *community engagement* from TMT as a whole. For example, one interviewee described how “*in every TMT role, we have to do community engagement*”; whereas others conflated other research activities, such as performing population census, to community engagement. Conceptually, community engagement could therefore be defined relatively easily, however, in practice, it was more difficult to demarcate it within specific activities at specific times. Staff also reported that they could have benefitted from further training, especially in the political context of different villages, in advocacy, and in concepts of community and what it means to engage a community.

### Targeted malaria treatment (and community engagement) in context

The project and the community engagement was entangled with the local social and cultural context, influenced, for example, by local ideas of hospitality, healing practices, socio-economic factors and practicality of conducting a clinical study.

#### Trust and local ideas of hospitality

Some villagers initially avoided meeting study staff in their village, preferring to *“watch and wait”*. However, later the same villagers often attended meetings, encouraged by village leaders or by staff conducting house-to-house visits. Others were repeatedly absent from the village because of lengthy periods of employment on rubber plantations, rice paddy fields or in the forest. For those who were present, it appeared important for villagers to see official government documentation approving the study, despite the fact they usually could not read the letters (due to illiteracy or the difference between Kayin and Burmese languages).

Repeat visits from staff members were necessary to build trust. Commensality—sitting round the same table and sharing traditional food—was also important. Staff participated in local social activities, such as the funeral of a villager (whose death was not TMT-related), and local festivals of touring opera singers. These activities also helped to build the trust that staff viewed as vital. Indeed, one staff member contended that trust needs establishing even before community engagement can begin:

*‘Only if [villagers] trust me, will we be able to engage with them…only after they trust us they will listen’.*

(S58, staff member).

Trust relationships were influenced by the concept of “*ah narr*”, whose translation to English proved problematic for translators. The term refers to an idea of avoiding social embarrassment, observing social niceties or being hospitable. Those not participating also told staff that they would have plenty of other participants—potentially another way of refusing the embarrassment of refusal. Furthermore, villagers showing agreement by nodding did not always imply real understanding and engagement:

*‘You know… the villagers have the habits of feeling bad in refusing to other people’ request. If you think it is good for them, they accept it’.*

(S53, staff member).

*‘We asked questions. But they just nodded their heads and did not say anything’.*

(S59, staff member).

Indeed, for staff, sometimes community engagement seemed successful in achieving adequate understanding of the purpose of TMT, but that it did not translate into willingness to participate in the project.

*‘So they accept when we give explanation about the TMT or why do we need to take drugs or something, they already accept and they appreciate for our activity in their village. But [when we ask], “Would you like to take [TMT study] drugs?” [they say] “No”’.*

(S60, staff member).

To develop the necessary trusting relationships, staff noted frequent contact with smaller groups was more important than fewer meetings of larger groups.

#### Religion, local healing practices, ethnicity and attitudes to biomedicine

Villagers followed a mix of religions: Buddhism predominated but other religions, including Christianity and a form of animism, were also reported. In terms of religion directly affecting the project, a small group of villagers said they would refuse to participate should any of the staff be Muslim; this reflected religious tensions at the national level.

Religion also featured in local healing practices, with local healers occasionally regarded as representing a religion. Villagers approached local healers to treat conditions of varying severity: one staff member described that some would even seek traditional medicine for broken limbs. Nonetheless, villagers also used biomedicine, including paracetamol and antibiotics, with some drugs administered intravenously. In spite of this, villagers’ use of faith-based local healing practice made some staff pessimistic about villagers’ readiness to take to Western medicines.

Interviewee: … *they also believe in traditional medicines and also traditional god….Yeah, traditional gods and spirits that they also believe, that most of them don’t believe in western medicines.*

Interviewer: … *when they are sick, what do they do?*

Interviewee: *Yeah, they drink water. And [the water] may be inspired by some monk or like old man [who says] “OK, drink that water, then you will feel well,” (laughs) yeah, they believe in that….* *(S58, staff member).*

Despite staff claims that local healing practices and western medicine were mutually exclusive, many villagers combined these approaches. Indeed, usage habits often depended on whether they could afford Western drugs. However, for staff trying to encourage villagers to participate in TMT, the faith-based approach to healing sometimes presented a challenge as they struggled with what they described as the baseless motives that some villagers gave for rejecting Western medicine.

Previous local programmes of mass filariasis treatment (which can lead to elephantiasis, a gross swelling of limbs or genitalia) generated rumours that the government were attempting ethnic cleansing via drug administration. Such fears also generated similar rumours about the safety of TMT. This accorded with many villagers’ life-histories, which included violent encounters with the Burmese military and presented a particular challenge for Burmese staff in the TMT team. Generally, however, inter-ethnic differences between other ethnicities (Kayin, Shan and Mon) in the study site did not seem to affect willingness to take part in TMT. Accordingly, community engagement was not adapted according to ethnicity. However, as an exceptional case, in one village, an ethnic subgroup, initially declined to participate as a group. Later in the study, however, staff reported that members of this group reconsidered their initial rejection.

#### Local healthcare infrastructure and socio-economic status

Villagers’ readiness to use biomedicine was illustrated by them seeking the help of VHWs, particularly those with whom they had developed rapport. Typically, they sought help to treat common fever and flu symptoms. They were often referred to MAM for more serious illness. Although there are regional hospitals in the local townships, where villagers could seek assistance for serious complaints, they were constrained by flooded transport routes in the rainy season and distances of up to 1.5 h by motorbike.

In light of the relatively limited local options of biomedical care, villagers were satisfied that their health was important to study staff. Indeed, villagers came to expect other healthcare intervention to be on offer when they attended the visiting TMT study clinics:

*‘We bring other mobile clinic drugs like many analgesic or antibiotic or others…This treatment also…we attract the villagers… They take TMT drugs. If we refuse… [if] we don’t have medicine….the villagers dislike [it]…’.*

(S57, staff member).

Furthermore, villagers’ satisfaction at seeing that TMT staff sought to improve community health tended to provoke discussions about improving social services more broadly, in terms of health and education infrastructure, for example.

Wealthy community members were however more able to access the available healthcare infrastructure. Village shopkeepers, for example, were part of the community in so far as they situated their shops in the village and profited from villagers’ purchases, but accessed healthcare in regional hospitals rather than through TMT. Villagers who worked in Thailand (and their relatives) were also sometimes wealthier and more educated. In addition to increasing their healthcare option, this also seemingly weakened their sense of belonging to the local community, and appreciation of the possible collective benefit of TMT. It was therefore harder for staff to attract these groups to participate in TMT, if relying on their sense of community or the access to medical care.

#### Ideas about malaria and decision-making

With a view to highlighting the benefits of TMT, community engagement activities sought to assess and increase villager understanding of the specific dangers of malaria. Villagers had general ways of referring to malarial symptoms, and used the term “forest sick” to describe fever, weakness and dizziness. Although people could link malaria-like illness with a location like the forest, they did not always know why the location made them ill. People could generally distinguish symptoms from diseases but sometimes malaria was seen as a symptom rather than a disease. This points to difficulties villages had in defining malaria as a distinct, serious disease.

Interviewer: *Have you had malaria before?*

Interviewee: *I do not know about the malaria. But I am always sick. I do not know what kinds of disease do I have in my body? May be I have got malaria, because, I am not healthy. I am not sure about my disease*.* (V08, villager)*.

However, villagers who had personal experience of malaria were able to recognize its seriousness and seek diagnosis and treatment from VHWs. Malaria prevention practices were uncommon or misunderstood, for example, staff explained that malaria could not be transmitted by drinking spring water and eating bananas. Long-lasting insecticide-treated bed nets had been distributed in some but not all target villages prior to TMT. Use of long-lasting insecticide-treated bed nets was limited due to hot night-time temperatures, even when mosquitoes were understood to carry disease.

Decisions about participating in TMT were, therefore, made in background of general understanding of malaria and some preoccupation about the illnesses. Decisions were not however necessarily made on an individual basis. In study villages, typically within families, fathers were engaged in subsistence farming, whereas mothers were charged with the day-to-day tasks of childcare at home. Fathers often could make decisions about TMT on behalf of the whole family, sometimes not participating themselves but putting forward the rest of the family for participation.

*‘So normally the father is the influential people in the family and [mostly] the fathers are the one who doesn’t take the TMT…the father says “I had never experienced malaria… but my wife and children they [have] experience[d it] so let them take. I don’t need to take” so he left at home’.*

(S54, staff member).

#### Community engagement within TMT

The project was subject to unavoidable time constraints; TMT was subject to several delays that reduced the time available to consult with key individuals about the optimal community engagement strategy. Staff met with representatives from each village, but meetings focused on the more urgent tasks of giving information and obtaining permission to start the study. Moreover, the study protocol stipulated limited periods for specific intensified community engagement activities, such as follow-up and monitoring of adverse events (after treatment rounds and before treatment teams moved onto other study villages, or returned to Yangon for debrief). The pressure of these timings, when combined with difficult topography and mobility issues, meant that staff had less time than they would have liked for community engagement.

Interviewer:

*How can we improve community engagement?*

Interviewee:

*We have take [more] time and also… the group discussion it is difficult. But for the.. like taking a census we have to go house to house. So for the census we had to take time I think, not only 1* *day, but about 2* *days or 3* *days, we go there and we discuss, we explain very well…*

(S63, staff member).

Also, although staff planned for formal evaluation of community engagement delivery (as evidenced by study protocol documents), in practice these time constraints complicated their documenting of community engagement activities.

A lack of detailed written procedures for how community engagement should be conducted meant that staff relied on verbal communication of the strategy. In addition, the intensity of the work in a tough rural region led to high staff turnover over the course of the study, and new staff were sometimes unaware of the community engagement activities conducted by previous staff. Staff suggested that community engagement would have benefitted from standard operating procedures. This implied that community engagement delivery would be (undesirably) variable if dependent on staff members’ individual approaches.

## Discussion

The findings illustrate the complexity of conducting TMT in an isolated setting with poor infrastructure and a recent history of conflict. To promote participation in the project, staff had to respond to the complex social context and adapt community engagement to emerging issues. For example, engaging with the community entailed dealing with armed groups, who, although not antagonistic to staff, required special attention to smooth the implementation of the project. It also required identifying influential community members who did not necessarily occupy the most obvious positions in the social hierarchy. Moreover, staff recognized that the frequency of interactions with community members was more important than the scale of the meetings. The nature of the community engagement was also influenced by the constraints on the wider project, particularly in terms of the available time. There was sometimes less clear separation of community engagement activities from those associated with the wider project.

Community engagement for TMT was hence entangled with the local social context and the wider project. Separating out its influence and assessing its effectiveness, regardless of the criteria used, raised metric/evaluation issues which others have also noted [22]. Nonetheless, levels of population coverage for the mass anti-malarial administration were higher than the estimated level necessary to potentially interrupt transmission of *P. falciparum* [8]. Therefore, using this indicator alone—and ignoring any wider impact of community engagement activities that came, for example, from providing health education—the community engagement benefitted the project.

In terms of undertaking community engagement, it is important to understand the dynamics of trust, taking into consideration local social and cultural particularities. Indeed, trust is recognized as particularly influential for community engagement and informed consent processes in biomedical research [14, 23], with some suggesting it offers benefits in fragmented communities [24]. In this study, local concepts of hospitality contributed to the building of trust, and villagers’ apparent acquiescence to TMT did not necessarily indicate that staff had built sufficient rapport (or that participants had understood the nature of the project). By contrast, a recent study in Vietnam indicated that receiving information about the MDA from district health workers rather than non-local staff improved coverage [14]. At the study site, where health infrastructure was limited, trust was founded not only on the immediate short-term provision of additional healthcare, but also through demonstrating a commitment to the local health needs. The trustworthiness of the project was challenged by rumours linked to a past filariasis MDA and some villagers took a “wait and see” approach, delaying participation. However, the community engagement—and the project as a whole—seemingly led them to overcome their concerns and ultimately participate at a later stage.

The high levels of participation in the mass anti-malarial administration also raises questions about the pessimism that some staff expressed regarding the response of villagers to Western medicine. Moreover, the medical care that TMT staff provided—as well as playing a role in engendering trust—was also in demand amongst villagers, and, in some instances, staff had to manage excessive demand and possible over-treatment. This highlights the need to avoid hasty judgements and generalizations about communities’ healing practices and how such practices might impact medical interventions. Indeed, the sub-groups that were most problematic for community engagement were the most educated and wealthy, whose links to the wider community were also less strong (a finding echoed by Kajeechiwa et al. [24]).

Given their importance to the *success* of TMT, understanding the local social and cultural context and a capacity for flexibility are, therefore, important considerations for community engagement training. In terms of learning during future projects, formal record-keeping may help to keep community engagement responsive to emerging issues. However, the logistical challenges encountered in recording community engagement activities and conducting formal learning activities during TMT suggest this risks over-burdening staff.

## Strengths and limitations

By examining community engagement within TMT’s social and cultural context, this article provides an overview of the factors that influenced attitudes and behaviours towards TMT in Kayin State, Myanmar. Using multiple data sources—interviews, photos (informal contributors to the analysis), study documents—and data collected independently by two members of the research team facilitated triangulation of findings and strengthened the resultant conclusions. Although the study shows that community engagement was important in TMT, it was not possible to determine a direct relationship between community engagement and population coverage.

Recording and monitoring each community engagement activity at the point of delivery was not possible because of time pressures and the need for a flexible and adaptive approach but details of the community engagement activities were collected from study staff. The villages were inaccessible to most of the authors and therefore extensive training and debriefing was carried out with the field worker. Moreover, because of the limited numbers of suitably qualified staff (experienced in qualitative health research) it was also not possible to recruit multiple field workers, which would have allowed further triangulation of the findings. Limited access to the study site also meant that some data collection (staff interviews) was retrospective because staff had been working in the field.

## Conclusion

Community engagement for TMT in Kayin State, Myanmar was entangled with the local social and cultural context as well as the implementation of the wider project. Although pre-planned in terms of approach and activities, community engagement was more fluid in practice, with staff responding to emerging issues and the heterogeneity of communities. Community engagement was hence also difficult to separate from the wider project activities and it was rather integral to all study staff’s work at the study sites. Because of the complex relationship between the local context and community engagement it is difficult to infer causality with regard to the success of the particular community strategy *per se*. Nonetheless, the project did record levels of population coverage for the mass anti-malarial administration above the projected level necessary to achieve interruption of *P. falciparum* transmission.
